# Special Issue “Polymeric Materials for Biomedical Applications, Drug and Gene Delivery”

**DOI:** 10.3390/ijms27062888

**Published:** 2026-03-23

**Authors:** Ilya E. Nifant’ev

**Affiliations:** 1 A.V. Topchiev Institute of Petrochemical Synthesis RAS, Leninsky Pr. 29, 116991 Moscow, Russia; ilnif@yahoo.com or inif@org.chem.msu.ru; 2 Chemistry Department, M.V. Lomonosov Moscow State University, Leninskie Gory 1–3, 119991 Moscow, Russia

An amazing variety of microstructures and molecular architectures of polymers, a vast range of approaches to their synthesis, and the diversity of the macromolecules of natural origin opens up tremendous opportunities for the search and implementation of polymeric materials in biomedicine and clinical practice [1,2,3,4,5]. Polymers can be rightfully called “21st century materials” towards biomedical applications, which is well illustrated by an exponential growth in the number of research papers and patents, presented in Figure 1 [6].

The brooks flow into the river, and I present here the contribution of colleagues from different countries to form the Special Issue *Polymeric Materials for Biomedical Applications, Drug and Gene Delivery*, which features a collection of seven articles. This Special Issue highlights six innovative studies and one critical review that encompass advances of polymers for various prospective directions in their biomedical use. I extend my sincere gratitude to the authors, peer reviewers, and editorial team whose contributions have made the preparation and publication of this Special Issue possible. Below is a brief summary of the papers included in this Issue, considering the different approaches to the choice and rationale of the scientific problem, research methodology, and results of the studies, along with their future prospects.

The first article of the Special Issue [7] describes a successful way to overcome toxicity of the promising X-ray contrast agents, Na_2_H_14_[{Re_6_Q_8_}(P(C_2_H_4_COO)_3_)_6_] (Q = S, Se), via PEGylation. In vitro (Hep-2 cells test) and in vivo (acute toxicity test in mice) revealed at least a two-fold decrease of toxicity in comparison with unmodified complexes; one of the PEGylated compounds was used for the visualization of blood vessels by angiography and computed tomography (rats). This investigation represents the further development of previous works [8,9,10,11] and has demonstrated a shining example of the biomedical use of PEGylation, in addition to the well-known regulation of hydrophilicity [12] and stealth effect [13].

The development of polymer vehicles for delivery of the complex of antiarrhythmic medication allapinin with monoammonium salt of glycyrrhizic acid was based on the design of polyelectrolite microcapsules [14]. These capsules, comprising polyallylamine and polystyrene sulfonate, showed reversible ability to adsorb and release the toxic drug, maintaining its therapeutic concentrations at physiological pH. In comparison with known vehicles for allapinin delivery [15,16,17,18,19,20], developed microcapsules have demonstrated a gradual release of the substance over a period of more than 24 h and a reduced release rate at physiological pH ~5.

The study at the interface of organic and bioorganic chemistries [21] was devoted to the synthesis of zwitterionic bridgehead boron heterocycles and their binding with calf thymus DNA. In comparison with known methods [22,23,24,25], more “green” and environmentally benign approaches to similar heterocycles were developed. The good intercalative properties of bridgehead boron heterocycles allow to consider them as lead compounds or drug carriers in the treatment of DNA-related diseases.

The fundamental issue of biocompatibility of biodegradable polymers [26] is an inflammatory response at the initial stage of the implant replacement by regenerating tissues. However, there is still no single marker in medicine for assessing the body’s reaction to the implantation of synthetic materials [27,28]. C-reactive protein (CRP) generally reflects inflammatory processes in various organs and systems [29]; its variant hs-CRP is a more sensitive marker [30]. In the study of Eisenach et al., presented in the Special Issue [31], the measurements of hs-CRP concentration in blood and morphometric studies were proposed and used to assess postsurgical effects in three-month experiments on subcutaneous implantation of poly(ε-caprolactone)/poly(trimethylene carbonate) (65:35 *w*/*w*) films (male Wistar rats). Inflammation occurred after 1 month after surgery, and the level of hs-CRP in the blood correlated with inflammatory activity in the tissues. It is noteworthy that a three-month period proved to be insufficient for the recession of inflammation. The method is certainly effective and can be used when evaluating the biocompatibility of other polymeric materials to minimize postoperative morbidity.

The development of synthetic bone substitutes is a heated topic of bone surgery and orthopedics, and biodegradable polymers hold a special place among prospective materials designed for this purpose. Poly(lactic acid) (PLA) represents FDA approved polyester, widely used in clinical practice, and further improvement of its biocompatibility is a relevant task [32,33]. An efficient way to modify the PLA surface via deposition of hydroxyapatite (HAp) [34] and dihexadecyl phosphate [35] as an interphase compatibilizer was proposed and studied by Prochaska and colleagues [36]. This work, presented in the Special Issue, was a continuation of a previous study of a two-component PLA/HAp composite [37]; the addition of compatibilizer improved the surface properties and biocompatibility of 3D-printed implants.

The last research article in the Special Issue [38] is also devoted to the development of synthetic bone substitutes, based on bioactive glasses (BGs) [39]. The formation of a 3D inorganic polymer network in the presence of potentially soluble fillers, followed by leaching of the filler, resulted in the formation of mesoporous BGs. The impact of the thermal BG treatment on loading efficiency and drug release, underexplored in previous works [40,41,42], was investigated in more detail in this study. New materials demonstrated fast biomineralization in vitro and high drug loading efficiency vs vancomycin, efficient medication for treating implant-related infections [43].

A comprehensive review [44], presented in the Special Issue, reflects the recent trend in the development of optical materials, based on biocompatible natural polymers, proteins and polysaccarides. In this review, the common description of polysaccaride biopolymers (cellulose, chitosan, and alginate) and proteins (silk fibroin, collagen, and gelatin) and a brief discussion of the key aspects of optical theory in biomedicine precedes a detailed and informative discussion of the methods of the preparation of optical devices. A critical assessment of the applicability of these products as optical waveguides and fibers, ocular implants, bioimaging, and diagnostic materials, concludes a review. It is noteworthy that, in this field, biodegradability is a disadvantage rather than an advantage, but this is offset by other merits of biobased optics. Important new data, generalizations, and conclusions presented in this review significantly supplement early review works [45,46,47,48].

Our scientific group is deeply involved in research of biodegradable polymers as composite components, orthopedic materials, and drug and gene delivery vehicles. Work on this Special Issue has greatly expanded our own ideas concerning the role and place of macromolecules in biomedicine, and has greatly contributed to our studies in this multifaceted area. We hope that the discoveries and insights presented and discussed in this Special Issue will contribute to further innovation and deeper interdisciplinary cooperation. This Special Issue serves not only as a collection of works; it is, rather, an invitation to a joint creative effort by scientists involved with the study of polymer materials and their diverse biomedical applications.

## Figures and Tables

**Figure 1:**
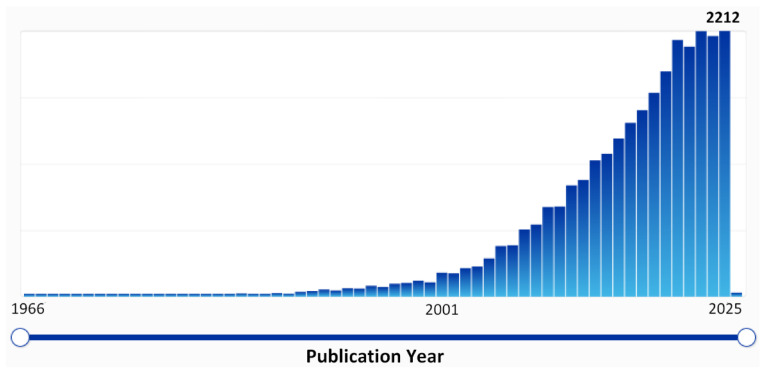
Publication statistics on the request “Polymer” AND “Biomedical” (Abstract/Keywords field, CAS SciFinder [6]).
